# Correlates of antenatal anxiety: smartphone use, depressive symptoms, and hypertensive disorders in a cross-sectional study in Southwest China

**DOI:** 10.3389/fmed.2025.1682499

**Published:** 2025-10-13

**Authors:** Tianzhi Guo, Yun Yu, Xia Wang, Yuqin Sun, Chao Yang

**Affiliations:** ^1^Department of Obstetrics, The Affiliated Hospital, Southwest Medical University, Luzhou, China; ^2^Department of Oral and Maxillofacial Surgery, The Affiliated Hospital, Southwest Medical University, Luzhou, China

**Keywords:** antenatal anxiety, smartphone use, depressive symptoms, hypertensive disorders, risk prediction

## Abstract

**Background:**

Antenatal anxiety is a prevalent yet underrecognized mental health condition with significant consequences for maternal and fetal outcomes. This study aimed to identify psychosocial and behavioral factors independently associated with antenatal anxiety among pregnant women in Southwest China.

**Methods:**

We conducted a retrospective cross-sectional study of 972 pregnant women consecutively attending routine antenatal care at a tertiary hospital in Southwest China (June 2022–June 2023). Eligible participants were ≥18 years with singleton pregnancy; women with pre-existing psychiatric disorders or major fetal anomalies were excluded. Anxiety and depressive symptoms were assessed using the Chinese versions of the GAD-7 and PHQ-9. Clinical, sociodemographic, and smartphone use data were obtained from electronic records and structured assessments. Multivariate logistic regression was used to identify independents of antenatal anxiety (GAD-7 ≥ 10), adjusting for relevant confounders. Model performance was evaluated using ROC curves, calibration plots, and a nomogram.

**Results:**

Antenatal anxiety was present in 51.4% of participants. Independent correlates factors included gestational age (OR = 0.94 per week, *p* = 0.002), junior high school education or below (OR = 1.81, *p* = 0.015), hypertensive disorders of pregnancy (OR = 2.06, *p* = 0.010), smartphone use >4 h/day (OR = 3.01, *p* < 0.001), nighttime use (OR = 2.26, *p* < 0.001), social networking as primary use (OR = 1.57, *p* = 0.013), and PHQ-9 score (OR = 1.31 per point, *p* < 0.001). The model showed strong predictive accuracy (AUC = 0.803) and good calibration.

**Conclusion:**

Smartphone overuse, nighttime use, social networking as the primary activity, depressive symptoms, hypertensive disorders, lower education level, and shorter gestational age are significant correlates of antenatal anxiety. Integrating digital behavior monitoring and mental health screening into routine prenatal care may help identify high-risk individuals for early intervention.

## Introduction

Antenatal anxiety, defined as persistent worry or tension during pregnancy, is common yet frequently overlooked. It has been associated with adverse obstetric and neonatal outcomes, including increased risks of preterm birth, low birthweight, and impaired neurodevelopment in early childhood (1, 2). Globally, the prevalence of antenatal anxiety symptoms is estimated at ~29%, with particularly high rates in low- and middle-income countries. In China, a recent meta-analysis reported a pooled prevalence of 17.4% among pregnant women (3–5). Despite its clinical importance, antenatal anxiety often goes undetected in routine practice, in part because reliable behavioral risk markers are lacking.

An increasing number of studies suggest that problematic smartphone use contributes to anxiety, depressive symptoms, and sleep disturbances in the general population. For example, a cross-sectional survey of 761 university students found that smartphone addiction was independently associated with higher levels of anxiety, depression, stress, and poor sleep quality (6). Similarly, a population-based study reported a dose–response relationship between smartphone overuse, anxiety severity, and reduced sleep quality (7). In pregnant women, excessive electronic device use—including prolonged screen exposure and mobile phone overuse—has been linked to greater psychological distress, particularly prenatal stress and depression. One path analysis study showed that smartphone use exceeding 8 h per day was associated with nearly twofold higher odds of depressive symptoms (adjusted OR ≈ 1.90–2.62) (8). Another cross-sectional study also confirmed significant correlations between problematic smartphone use and pregnancy-related depression (9). However, direct evidence connecting smartphone behaviors to antenatal anxiety remains scarce, especially in low- and middle-income countries such as China.

In China, existing research has primarily focused on sociodemographic and obstetric correlates of antenatal anxiety, as well as the psychological impact of COVID-19–related stressors. Maternal education, parity, and pregnancy complications have consistently emerged as key correlates in cross-sectional studies (10). During the pandemic, heightened levels of antenatal anxiety and depression have been associated with stressors such as social isolation, fear of infection, and disruptions to antenatal care (11). Nevertheless, few studies have examined behavioral risk factors such as the duration of smartphone use, nighttime usage patterns, or the primary purpose of smartphone engagement (e.g., social networking versus information seeking)—factors that may reflect emotional regulation strategies or sleep disruption (9, 12, 13).

Guided by a biopsychosocial stress–vulnerability framework, we propose that problematic smartphone use may exacerbate antenatal anxiety through several plausible pathways. First, bedtime and prolonged screen exposure can heighten cognitive arousal, disrupt circadian rhythms, and impair sleep quality—well-established antecedents of anxiety. Second, predominant use for social networking may intensify social comparison and exposure to negative content, reinforcing rumination and emotional dysregulation. A complementary mechanism is attentional capture and displacement of restorative activities, such as physical exercise or face-to-face social support, thereby reducing coping resources. These theoretical considerations informed our hypotheses that daily duration, nighttime use, and social networking as the primary purpose of smartphone use would independently predict antenatal anxiety risk.

Problematic smartphone use often co-occurs with depressive symptoms, which have been consistently associated with antenatal anxiety. In a cross-sectional study of 665 women in early pregnancy in China, both longer daily screen time and higher smartphone addiction scores were independently associated with greater depressive symptom severity on the PHQ-9 (adjusted OR per hour = 1.09; per addiction score point = 1.11; *p* < 0.05) (9). Longitudinal evidence from Chinese young adults further indicates that smartphone addiction not only worsens depressive symptoms but also aggravates sleep disturbances over time (14).

In addition to behavioral variables, hypertensive disorders of pregnancy (HDP)—such as gestational hypertension and preeclampsia—and lower educational attainment have also been independently linked with heightened antenatal psychological distress. A recent Chinese cohort study reported significantly higher rates of anxiety among pregnant women diagnosed with HDP compared to normotensive controls (10). Furthermore, large-scale urban surveys have consistently shown that lower maternal education is significantly associated with antenatal anxiety and depression, even after adjusting for socioeconomic and obstetric confounders (11). Whether smartphone-related behaviors contribute additional predictive value beyond these established clinical and psychosocial risk factors remains unclear.

To address these gaps, we conducted a retrospective cross-sectional study among pregnant women in Southwest China. The primary objective was to examine behavioral and psychosocial factors associated with antenatal anxiety, with a particular focus on smartphone use characteristics. We hypothesized that daily duration, nighttime usage, and use primarily for social networking would be independently associated with anxiety risk, even after adjusting for depressive symptoms, HDP, educational level, and gestational age.

## Methods

### Study design and setting

This retrospective cross-sectional study was conducted at a tertiary-grade A hospital in Southwest China between June 2022 and June 2023. The target population comprised pregnant women attending routine antenatal care during the study period. All data were sourced from standardized electronic medical records and structured nursing assessments collected as part of routine clinical care. The study aimed to explore psychosocial and behavioral determinants of antenatal anxiety within a real-world clinical setting.

### Participants

A total of 972 pregnant women were consecutively enrolled. Inclusion criteria were: (1) age ≥18 years, (2) singleton pregnancy, and (3) complete data on both psychological assessments (GAD-7 and PHQ-9) and relevant clinical variables. Exclusion criteria included: (1) documented psychiatric disorders diagnosed prior to pregnancy (based on ICD-10 codes), (2) cognitive or language barriers that prevented questionnaire completion, and (3) major fetal anomalies or intrauterine fetal demise as confirmed by ultrasound. All eligible cases within the study timeframe were included without additional filtering. Data extraction followed a standardized protocol, and missing data handling is detailed in the Statistical Analysis section.

### Psychological measures

Psychological status was assessed using the validated Chinese versions of the Generalized Anxiety Disorder-7 (GAD-7) and the Patient Health Questionnaire-9 (PHQ-9). Questionnaires were completed independently by participants in a quiet consultation room, with trained obstetric nurses available to clarify procedural questions without offering interpretation or influencing responses. Participants were instructed to respond based on their experience during the prior 2 weeks. A GAD-7 score ≥10 was used to define clinically significant anxiety, consistent with prior validation studies in pregnant Chinese women (15). To examine the robustness of this threshold, we also conducted a sensitivity analysis using an alternative cutoff of ≥8, which has been recommended in some perinatal studies to improve sensitivity. Results of this analysis are presented in Supplementary Table S1. The *a priori* choice of the ≥10 threshold prioritized specificity and comparability with prior obstetric studies, including validation work in pregnant Chinese women (15); PHQ-9 was retained as a continuous severity indicator. PHQ-9 scores were treated as continuous measures of depressive symptoms.

### Sociodemographic and clinical variables

Demographic variables included maternal age, education level, employment status, and parity. Education was categorized as: (1) junior high school or below, (2) high school/vocational school, and (3) college degree or higher. Parity was defined as primiparous (no previous delivery) or multiparous (≥1 previous delivery). Gestational age was based on first-trimester ultrasound-confirmed dating and recorded as completed weeks at the time of survey completion. Pregnancy complications were obtained from physician-documented diagnoses and categorized as: (1) none, (2) gestational diabetes mellitus (GDM), (3) hypertensive disorders of pregnancy (HDP), or (4) other (e.g., intrahepatic cholestasis).

### Smartphone use assessment

Smartphone-related variables were collected using a standardized, structured questionnaire designed by the obstetric nursing team. Daily usage duration was self-reported and categorized as: <2 h, 2–4 h, or >4 h. Nighttime smartphone use was defined as device use within 1 h before sleep. Participants were also asked to select their primary smartphone use purpose from three mutually exclusive options: information seeking, social networking, or entertainment/gaming. All definitions and coding schemes were finalized before analysis to ensure standardization. To mitigate recall bias, nurses used standardized prompts and anchored the assessment to typical weekdays and weekends in the last 2 weeks; duration was captured in predefined categories (<2, 2–4, >4 h) and nighttime use was defined explicitly as within 1 h before sleep.

### Data quality control and bias mitigation

Data were independently extracted by two trained researchers using a structured extraction template. A double-entry verification system was implemented, with discrepancies resolved through consensus or review by a senior investigator. Prior to full data collection, a pilot extraction of 50 cases was conducted, yielding a Cohen’s kappa of 0.92, indicating high inter-rater reliability. Missing data were minimal (<2%) and assessed using Little’s MCAR test, which confirmed that missingness was completely at random (*p* > 0.05). Therefore, complete-case analysis was applied. To reduce information bias, all clinical variables were collected prior to knowledge of anxiety status. Psychological questionnaires were administered in a standardized, non-directive environment. Inclusion and exclusion criteria were prespecified to minimize selection bias.

### Statistical analysis

All statistical analyses were performed using SPSS version 26.0 (IBM Corp., Armonk, NY, United States) and R version 4.2.1 (R Foundation for Statistical Computing, Vienna, Austria). Two-tailed *p*-values <0.05 were considered statistically significant unless otherwise specified. Baseline characteristics were summarized using means ± standard deviations (SD) for continuous variables and frequencies (%) for categorical variables. Between-group comparisons were made using independent-samples t-tests and chi-square or Fisher’s exact tests as appropriate. Univariate logistic regression was conducted to evaluate factors associated with antenatal anxiety (GAD-7 ≥ 10). Variables with *p* < 0.10 or considered clinically relevant were entered into the multivariate logistic regression model using the enter method. Categorical variables (e.g., education level, smartphone use categories) were dummy-coded with the lowest-risk category as reference. PHQ-9 scores were entered as continuous variables. PHQ-9 was modeled per 1-point increase to preserve information and interpretability; importantly, logistic regression does not assume normality of the covariates. Multicollinearity was assessed using variance inflation factors (VIF), and all included variables had VIF < 2.5. To minimize overfitting, we limited the number of parameters in the multivariable model. With 500 events (anxiety cases) and 9 regression coefficients (gestational age; two education dummies; HDP; two duration dummies for daily smartphone use; nighttime use; social networking; and PHQ-9), the events-per-variable (EPV) was ≈55, which comfortably exceeds the conventional ≥10 rule of thumb. Internal validation was performed using 1,000 bootstrap resamples. Model discrimination was evaluated by the area under the receiver operating characteristic curve (AUC), and calibration was assessed using the Hosmer–Lemeshow test and a calibration curve based on 1,000 bootstrap resamples. A nomogram was developed from the final model to allow individualized risk estimation.

## Results

### Baseline characteristics of participants

Among the 972 pregnant women included in this study, 500 (51.4%) met the diagnostic threshold for anxiety (GAD-7 score ≥10), while 472 (48.6%) did not. As shown in Table 1, participants with anxiety had a significantly lower mean gestational age compared to those without anxiety (30.9 ± 5.2 vs. 31.6 ± 5.0 weeks, *p* = 0.041). The prevalence of lower education levels was significantly higher in the anxiety group (*p* = 0.019), with 15.2% having junior high school education or below versus 8.9% in the non-anxiety group. Hypertensive disorders of pregnancy (HDP) were more common among anxious participants (11.0% vs. 5.5%, *p* = 0.027). Notably, those with anxiety reported significantly longer daily smartphone use (*p* < 0.001), higher rates of nighttime usage (82.2% vs. 67.8%, p < 0.001), and a greater likelihood of using smartphones primarily for social networking rather than information seeking (*p* = 0.006). PHQ-9 scores, reflecting depressive symptoms, were also significantly elevated in the anxiety group (8.2 ± 4.3 vs. 3.9 ± 2.7, *p* < 0.001).

**Table 1:** Baseline characteristics of participants by anxiety status (*N* = 972).

Characteristic	No anxiety (*n* = 472)	Anxiety (*n* = 500)	*p*-value
Age, years, mean ± SD	29.4 ± 4.7	29.9 ± 4.9	0.078
Gestational age, weeks, mean ± SD	31.6 ± 5.0	30.9 ± 5.2	0.041*
Education level, *n* (%)			0.019*
Junior high or below	42 (8.9)	76 (15.2)	
High school/technical	124 (26.3)	140 (28.0)	
College or above	306 (64.8)	284 (56.8)	
Employment status, *n* (%)			0.203
Employed	368 (77.9)	378 (75.6)	
Unemployed	104 (22.1)	122 (24.4)	
Parity, *n* (%)			0.086
Primiparous	304 (64.4)	308 (61.6)	
Multiparous	168 (35.6)	192 (38.4)	
Pregnancy complications, *n* (%)			0.027*
None	354 (75.0)	337 (67.4)	
GDM	78 (16.5)	86 (17.2)	
HDP	26 (5.5)	55 (11.0)	
Other	14 (3.0)	22 (4.4)	
Daily smartphone use, *n* (%)			<0.001*
<2 h	102 (21.6)	56 (11.2)	
2–4 h	240 (50.8)	182 (36.4)	
>4 h	130 (27.5)	262 (52.4)	
Nighttime smartphone use, *n* (%)			<0.001*
No	152 (32.2)	89 (17.8)	
Yes	320 (67.8)	411 (82.2)	
Main smartphone use purpose, *n* (%)			0.006*
Information seeking	238 (50.4)	208 (41.6)	
Social networking	132 (28.0)	190 (38.0)	
Entertainment/gaming	102 (21.6)	102 (20.4)	
PHQ-9 score, mean ± SD	3.9 ± 2.7	8.2 ± 4.3	<0.001*

Values are presented as mean ± standard deviation or number (%). GDM, gestational diabetes mellitus; HDP, hypertensive disorders of pregnancy; PHQ-9, Patient Health Questionnaire-9. Anxiety status was defined as GAD-7 score ≥10. *p*-values were calculated using the independent-samples *t*-test for continuous variables and *χ*^2^ test for categorical variables. The primary purpose of smartphone use was self-reported by participants based on a single-choice nursing assessment. *indicates statistical significance at *p* < 0.05.

### Univariate analysis of factors associated with anxiety

The results of univariate logistic regression analysis are presented in Table 2. Several variables showed significant associations with anxiety. Increased gestational age was modestly protective (OR = 0.95, 95% CI: 0.92–0.98, *p* = 0.004), while lower education levels, particularly junior high school or below, were associated with elevated anxiety risk (OR = 1.92, 95% CI: 1.26–2.91, *p* = 0.002). HDP was a strong correlate of anxiety (OR = 2.23, 95% CI: 1.36–3.65, *p* = 0.002). Compared to <2 h of smartphone use per day, 2–4 h (OR = 1.55, *p* = 0.031) and >4 h (OR = 3.44, *p* < 0.001) were both significantly associated with anxiety. Nighttime smartphone use (OR = 2.41, *p* < 0.001) and using smartphones mainly for social networking (OR = 1.69, *p* = 0.002) also increased the likelihood of anxiety. Furthermore, each one-point increase in PHQ-9 score was associated with a 34% increase in anxiety risk (OR = 1.34, 95% CI: 1.27–1.42, *p* < 0.001).

**Table 2:** Univariate logistic regression analysis of factors associated with anxiety among pregnant women (*N* = 972).

Variable	OR (95% CI)	*p*-value
Age ≥35 years (yes vs. no)	1.28 (0.90–1.82)	0.167
**Gestational age (per week increase)**	**0.95 (0.92–0.98)**	0.004*
Education level (ref: college or above)		
**Junior high or below**	**1.92 (1.26–2.91)**	0.002*
High school/technical	1.22 (0.89–1.66)	0.216
Employment status (unemployed vs. employed)	1.14 (0.85–1.52)	0.384
Multiparous (vs primiparous)	1.13 (0.87–1.48)	0.361
Pregnancy complications (ref: none)		
GDM	1.06 (0.75–1.49)	0.752
**HDP**	**2.23 (1.36–3.65)**	0.002*
Other	1.49 (0.72–3.06)	0.275
Daily smartphone use (ref: <2 h)		
**2–4 h**	**1.55 (1.04–2.31)**	0.031*
**> 4 h**	**3.44 (2.30–5.14)**	<0.001*
**Nighttime smartphone use (yes vs no)**	**2.41 (1.75–3.34)**	<0.001*
Main smartphone use purpose (ref: information seeking)		
**Social networking**	**1.69 (1.22–2.35)**	0.002*
Entertainment/gaming	1.21 (0.83–1.76)	0.317
**PHQ-9 score (per 1-point increase)**	**1.34 (1.27–1.42)**	<0.001*

OR, odds ratio; CI, confidence interval; GDM, gestational diabetes mellitus; HDP, hypertensive disorders of pregnancy; PHQ-9, Patient Health Questionnaire-9. Anxiety was defined as a Generalized Anxiety Disorder-7 (GAD-7) score ≥10. All variables were entered individually into binary logistic regression models. Variables with *p* < 0.05 are marked with*; bold text indicates statistically significant variables.

### Multivariate analysis of independent correlates of anxiety

Multivariate logistic regression identified several independent correlates of anxiety, as shown in Table 3. Gestational age remained a protective factor (adjusted OR = 0.94, 95% CI: 0.91–0.98, *p* = 0.002). Participants with junior high school education or below had significantly higher odds of anxiety (adjusted OR = 1.81, *p* = 0.015). HDP independently predicted anxiety (adjusted OR = 2.06, *p* = 0.010). Daily smartphone use exceeding 4 h was strongly associated with anxiety (adjusted OR = 3.01, 95% CI: 1.95–4.66, *p* < 0.001), and even moderate use (2–4 h) showed borderline significance (adjusted OR = 1.49, *p* = 0.049). Nighttime use of smartphones (adjusted OR = 2.26, *p* < 0.001) and primary use for social networking (adjusted OR = 1.57, *p* = 0.013) were also significant. In the multivariate model, PHQ-9 score showed a strong association with anxiety (adjusted OR = 1.31 per point increase, *p* < 0.001). The model demonstrated acceptable discrimination (AUC = 0.803) and good calibration (Hosmer–Lemeshow test *p* = 0.49). In a sensitivity analysis using the alternative GAD-7 cutoff of ≥8, the overall direction and approximate magnitude of associations were consistent with the main analysis (Supplementary Table S1). The only deviation was for daily smartphone use of 2–4 h (vs < 2 h), which attenuated to borderline significance (adjusted OR ≈ 1.44, 95% CI ≈ 0.99–2.10; *p* = 0.056), while the >4-h category remained robustly associated with anxiety.

**Table 3:** Multivariate logistic regression analysis of factors independently associated with anxiety in pregnant women (*N* = 972).

Variable	Adjusted OR (95% CI)	*p*-value
**Gestational age (per week increase)**	**0.94 (0.91–0.98)**	0.002*
Education level (ref: college or above)		
**Junior high or below**	**1.81 (1.12–2.91)**	0.015*
High school/technical	1.24 (0.87–1.75)	0.230
**HDP (vs none)**	**2.06 (1.19–3.58)**	0.010*
Daily smartphone use (ref: <2 h)		
**2–4 h**	**1.49 (1.00–2.25)**	0.049*
**> 4 h**	**3.01 (1.95–4.66)**	<0.001*
**Nighttime smartphone use (yes vs no)**	**2.26 (1.58–3.23)**	<0.001*
**Main use: social networking (vs information seeking)**	**1.57 (1.10–2.23)**	0.013*
**PHQ-9 score (per 1-point increase)**	**1.31 (1.23–1.39)**	<0.001*

OR, odds ratio; CI, confidence interval; HDP, hypertensive disorders of pregnancy; PHQ-9, Patient Health Questionnaire-9. Anxiety was defined as a GAD-7 score ≥10. Multivariate logistic regression included all variables with *p* < 0.1 in univariate analysis or considered clinically relevant. Variables with *p* < 0.05 are marked with*; bold text indicates independent variables with statistical significance.

### Model performance and visualization

**Figure 1:**
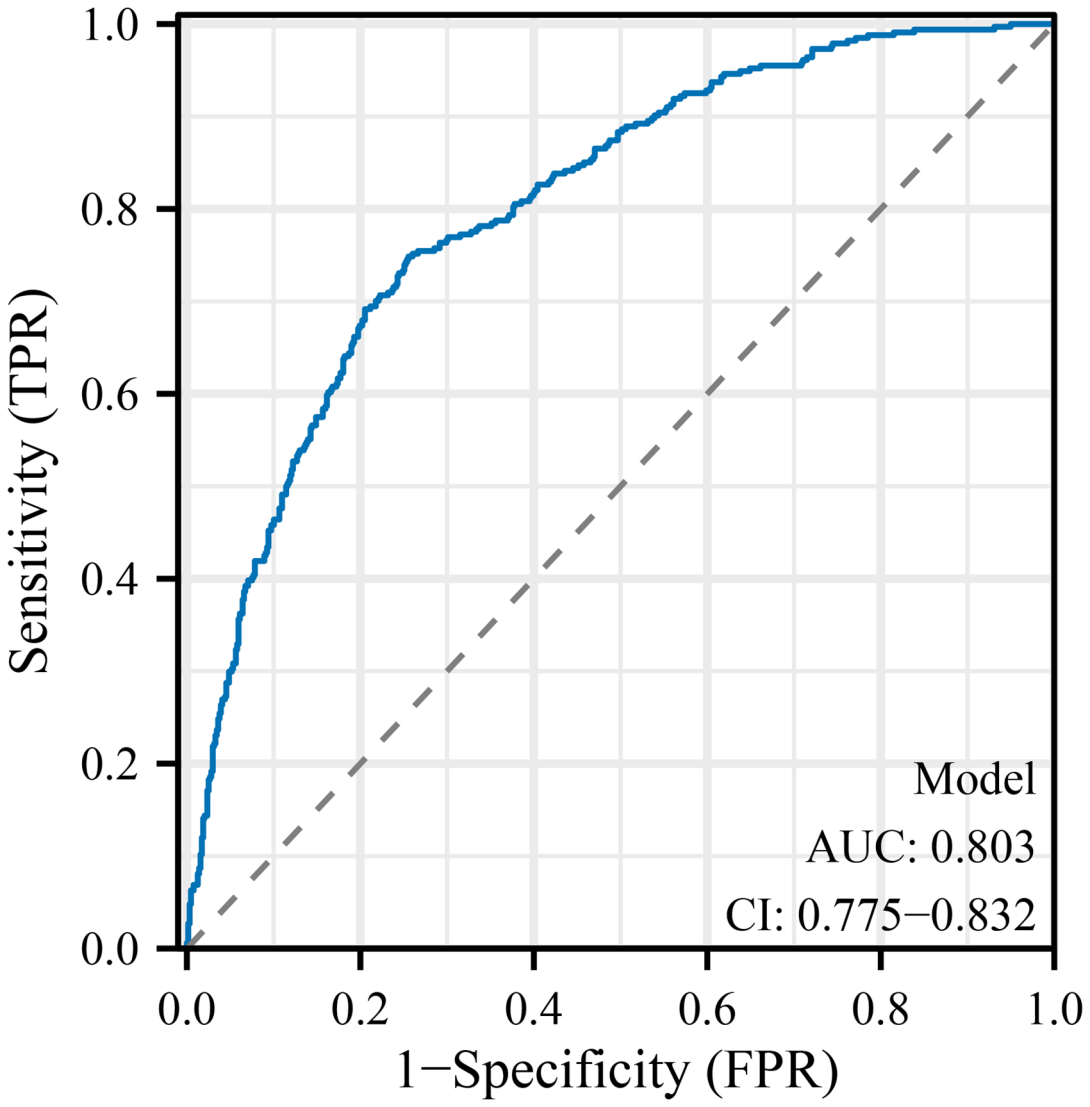
Receiver operating characteristic (ROC) curve for the multivariate logistic regression model predicting anxiety in pregnant women. The ROC curve demonstrates the discriminative ability of the final multivariate logistic regression model for predicting anxiety, defined as a GAD-7 score ≥10. The area under the curve (AUC) is 0.803, with a 95% confidence interval of 0.775–0.832, indicating good predictive performance. Among all factors, smartphone overuse (>4 h/day), nighttime use, social networking, PHQ-9 score, and HDP contributed most substantially to the model’s discriminative ability.

**Figure 2:**
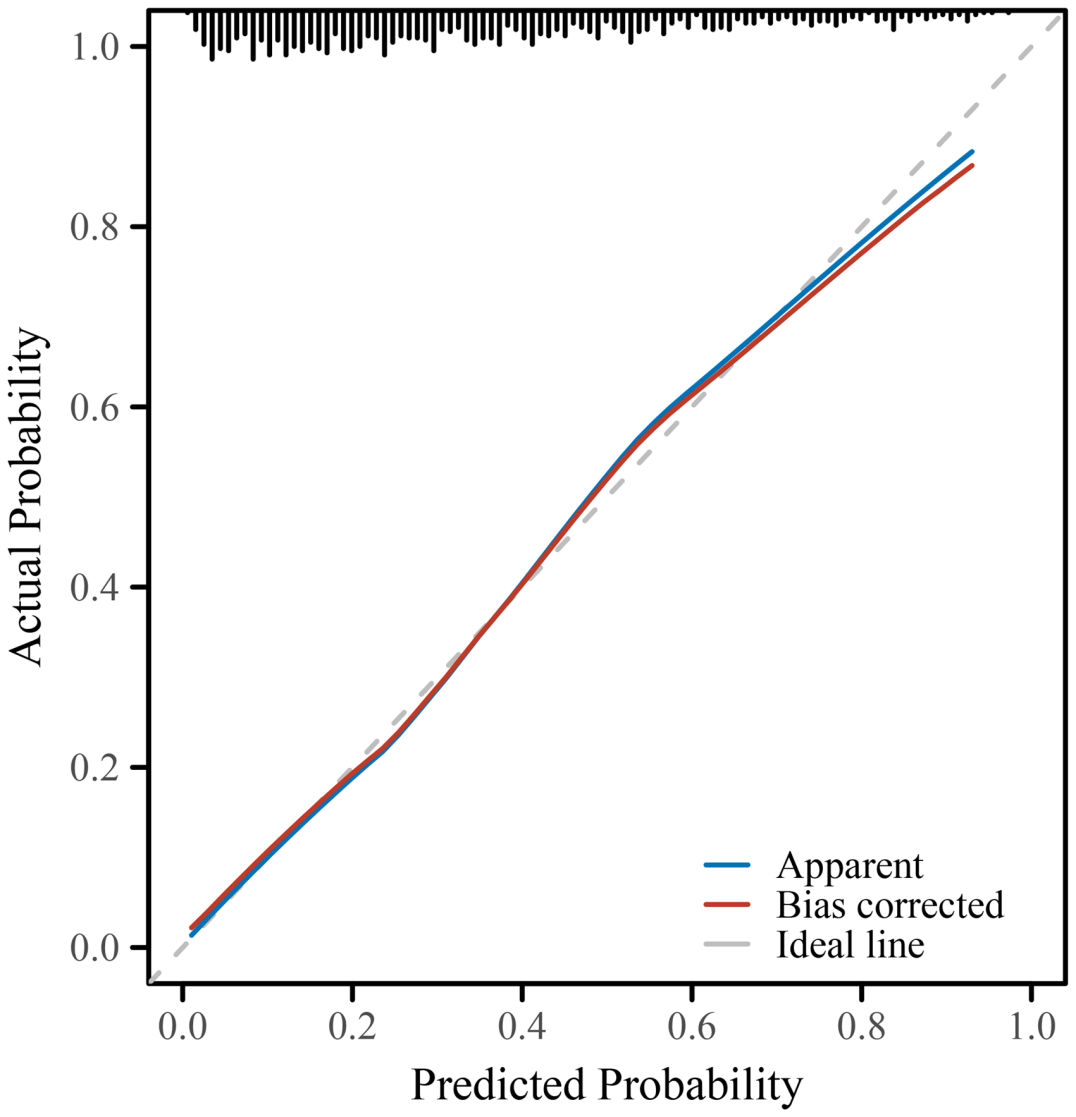
Nomogram for predicting the probability of anxiety during pregnancy. The nomogram was developed based on the multivariate logistic regression model and includes factors such as gestational age, education level, hypertensive disorders of pregnancy (HDP), smartphone use duration, nighttime smartphone use, social media use, and PHQ-9 score. To estimate individual anxiety risk, draw a vertical line from each variable to the “Points” axis, sum the total points, and locate the corresponding predicted risk on the “Risk” axis. Smartphone overuse (>4 h/day), nighttime use, PHQ-9 score, and HDP emerged as the most influential factors in the nomogram.

**Figure 3:**
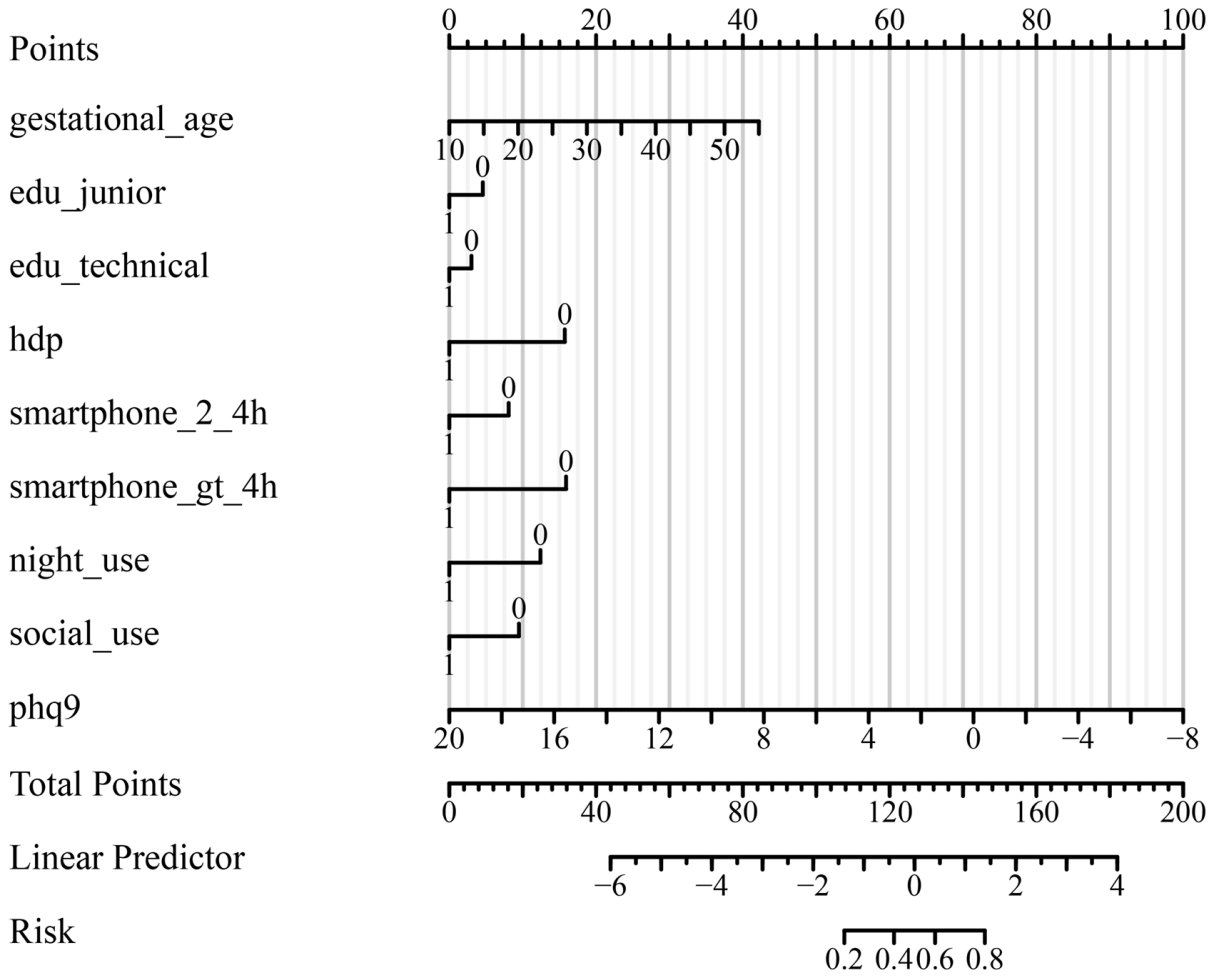
Calibration curve for the multivariate logistic regression model. The calibration curve assesses the agreement between the predicted probability and the observed incidence of anxiety in pregnant women. The ideal line represents perfect calibration. The bias-corrected and apparent curves indicate that the model is well calibrated across the range of predicted probabilities, with minimal deviation from the ideal. Calibration remained strong even for women with high-risk profiles (e.g., smartphone overuse, nighttime use, high PHQ-9 scores, and HDP).

The predictive performance of the final model is illustrated in Figure 1, with an area under the ROC curve (AUC) of 0.803 (95% CI: 0.775–0.832), indicating good discriminative ability. A nomogram was constructed to visualize the relative contribution of each correlate to anxiety risk (Figure 2). Variables such as smartphone overuse, nighttime use, social media usage, and depressive symptoms contributed heavily to total risk scores. The calibration plot in Figure 3 showed strong agreement between predicted and observed probabilities across the range of risk estimates, with minimal deviation from the ideal line, confirming the model’s reliability. Consistent with these results, the multivariable model’s AUC was 0.803 (95% CI: 0.775–0.832) based on ROC analysis with bootstrap internal validation.

## Discussion

This cross-sectional study identifies several factors associated with antenatal anxiety among pregnant women in Southwest China, including excessive smartphone use (especially >4 h/day), nighttime usage, social networking as the main activity, depressive symptoms, hypertensive disorders of pregnancy (HDP), and lower educational attainment. These results not only reinforce previous findings from general adult populations regarding the psychological risks associated with problematic smartphone use (6, 15), but also offer novel insights by quantifying these associations in a pregnant population—an area that remains underexplored, particularly in middle-income countries such as China. Notably, studies from diverse contexts—including Europe, Latin America, and Africa—have also reported associations between excessive digital device use and perinatal mental health concerns, suggesting that our findings may reflect a broader global phenomenon (16–18). Strengthening cross-cultural evidence is essential to contextualize digital behavior as a modifiable risk factor in maternal care worldwide.

Beyond observational studies, digital health and behavioral intervention research has also explored the role of mobile or app-based approaches in perinatal and related populations. For example, Chiou and colleagues have reported case studies of app-based interventions for postpartum depression and meditation training (19, 20), as well as experimental studies on the psychological effects of loving-kindness meditation delivered through short video applications (21, 22). In addition, Chen and colleagues examined the effects of animation and app design features on comprehension and user experience in healthcare contexts (22, 23). Collectively, these studies underscore the broader potential of digital platforms to influence mental health and health-related behaviors, complementing our focus on smartphone use as a correlate of antenatal anxiety.

Our finding that over half of participants (51.4%) screened positive for clinically significant anxiety is substantially higher than the pooled national estimate of 17.4% (5), and may reflect contextual or methodological differences. First, the study site is a large tertiary care center that likely receives higher-risk referrals, possibly over-representing psychologically vulnerable women. Second, our use of GAD-7 with a cut-off of ≥10 is highly specific and validated in pregnant Chinese women (15), yet may yield higher estimates in late second or third trimester samples, as has been reported elsewhere (24). The high prevalence observed (51.4%) may also partly reflect selection bias, as the tertiary hospital setting could overrepresent clinically vulnerable women. In addition, while we applied the GAD-7 cut-off of ≥10—validated in Chinese pregnant women (15)—this relatively strict threshold prioritizes specificity and may yield higher prevalence estimates compared with studies using lower thresholds (e.g., ≥8). These factors should be considered when interpreting the prevalence rate in our sample. Third, data collection occurred during the COVID-19 recovery period, a time when psychological distress among pregnant women remained elevated even after the peak of the pandemic in China. For instance, a cohort in Shanghai showed that depressive symptoms increased by 12.3% (from 35.4 to 47.7%), and this elevated negative mood persisted despite easing public health restrictions (25). Unfortunately, because precise visit dates were not consistently available in our research dataset, we were unable to adjust for pandemic timing as a covariate. Thus, residual pandemic-related stressors may still have acted as unmeasured confounders. Future studies with detailed temporal information (e.g., trimester-specific enrollment timing or direct indicators of pandemic stress) are warranted to clarify these contextual influences.

We examined multiple facets of smartphone use—daily duration, nighttime engagement, and primary purpose. This multidimensional assessment revealed consistent, independent associations between problematic smartphone use and antenatal anxiety, even after controlling for depression and obstetric risk factors. These findings align with prior literature showing that nighttime smartphone use disrupts circadian rhythms and sleep quality; for example, bedtime device use has been linked to increased pre-sleep cognitive arousal and poorer sleep (26). Likewise, predominant use for social networking may expose women to distressing content and social comparison, thereby reinforcing rumination and heightening anxiety symptoms (27, 28).

These findings can be interpreted within the stress–vulnerability framework, where behavioral exposures interact with psychosocial risk to heighten anxiety. The strongest associations observed for >4 h/day and nighttime use are consistent with a sleep-disruption pathway, whereas the effect of social networking aligns with a social-comparison and rumination pathway. Although our study was not designed to test mediation or moderation, the convergence of these behavioral dimensions strengthens the plausibility of these mechanisms and highlights potentially modifiable targets for intervention. It is important not to conflate the magnitude of an odds ratio for an individual factor with overall model discrimination. The OR of 3.01 for >4 h/day reflects a strong association for that exposure, whereas the AUC summarizes the combined performance of the included factors across decision thresholds. An AUC around 0.80 is generally considered good discrimination for complex psychosocial outcomes, indicating meaningful—but not perfect—predictive value consistent with the multifactorial nature of antenatal anxiety.

Depressive symptoms, measured via PHQ-9, were the most powerful continuous factor associated with antenatal anxiety—a finding consistent with contemporary evidence demonstrating high comorbidity of depression and anxiety among pregnant women (29, 30). While depressive symptoms may mediate part of the smartphone–anxiety link, our multivariate results suggest that smartphone behavior itself contributes uniquely to anxiety risk. Nevertheless, we acknowledge that smartphone overuse and depressive symptoms may partly reflect overlapping constructs, such as maladaptive avoidance or emotion regulation difficulties. Although our findings demonstrate that both variables retained independent predictive value in the multivariate model, the current analysis did not formally test mediation or moderation pathways. Future studies should employ longitudinal or structural equation modeling approaches to clarify whether depressive symptoms mediate the effect of problematic smartphone use on anxiety, or whether education level or gestational stage moderates these associations.

We found that women with hypertensive disorders of pregnancy (HDP) had approximately twice the odds of antenatal anxiety, consistent with recent evidence of heightened psychological distress in pregnancies complicated by gestational hypertension or preeclampsia (31). The predictive value of HDP remained significant even after controlling for gestational age and mood symptoms, underscoring its independent psychosomatic burden. Lower educational attainment was also associated with greater anxiety risk, likely reflecting both reduced access to health information and increased psychosocial stressors. Notably, smartphone-related variables retained significance after adjustment for education, indicating they are not simply proxies for socioeconomic status.

This study has several limitations that warrant careful consideration. First, its cross-sectional design precludes any determination of temporal or causal relationships. Although we observed a dose–response association between smartphone use and anxiety, it remains plausible that anxious individuals may increase smartphone engagement as a maladaptive coping strategy. Indeed, existing evidence suggests a potential bidirectional relationship, where problematic smartphone use may exacerbate anxiety symptoms, while heightened anxiety may, in turn, drive excessive device engagement as a form of avoidance or reassurance-seeking (14). Future longitudinal or ecological momentary assessment studies are warranted to clarify directionality. Longitudinal or ecological momentary assessment (EMA) studies are needed to disentangle directionality and capture dynamic interactions over time. Second, the study was conducted at a single tertiary hospital in Southwest China, which may limit the generalizability of findings to other regions, rural populations, or different healthcare levels. In particular, rural populations, women from lower socioeconomic backgrounds, and those receiving care in non-tertiary settings may exhibit distinct smartphone use patterns, mental health stressors, and barriers to digital health interventions. Therefore, external validation in such populations is warranted to ensure broader applicability of our findings. Because tertiary centers often receive higher-risk referrals, our sample may overrepresent clinically vulnerable women, leading to a higher observed prevalence of anxiety. Future multicenter studies including community and rural populations are needed to validate the generalizability of our findings. The exclusion of individuals with pre-existing psychiatric disorders may also introduce selection bias by underrepresenting those most vulnerable to antenatal anxiety, potentially leading to an underestimation of overall prevalence. This decision was made to reduce heterogeneity and confounding by chronic psychiatric illness; however, future research should include these populations to more accurately reflect the burden of antenatal anxiety in clinical practice. We also acknowledge that limiting recruitment to a single tertiary hospital may restrict representativeness. This site was chosen as it is the largest referral center in Southwest China with standardized obstetric and psychological assessment protocols, which ensured data quality and consistency. However, future studies should extend recruitment to community hospitals and rural settings to enhance generalizability. Third, although we used validated screening instruments (GAD-7 and PHQ-9), these tools are not diagnostic, and self-reported measures may be susceptible to recall and social desirability biases. We did not evaluate alternative functional forms for PHQ-9 (e.g., categorical thresholds or restricted cubic splines); future work should examine potential non-linear or threshold effects. For smartphone behavior specifically, measurement relied on self-report, which may be subject to recall bias. Although the use of categorical duration bands and an operational definition of nighttime use helped standardize reporting, objective digital-tracking metrics (e.g., system screen-time logs or app-based monitoring) would further strengthen measurement validity and are a priority for future studies. Notably, the GAD-7 cutoff of ≥10 has high specificity but may misclassify cases with subthreshold or atypical anxiety symptoms. To address this, we conducted a sensitivity analysis with a lower threshold (≥8), which produced substantively similar findings (Supplementary Table S1), thereby supporting the robustness of our conclusions. Nevertheless, trimester-specific validation studies remain warranted to determine optimal cut-points for perinatal screening in China. Fourth, several potentially important confounders were not assessed, including sleep quality, social support, marital conflict or satisfaction, history of intimate partner violence, family psychiatric history, and adverse childhood experiences—all of which are known contributors to perinatal mental health. The omission of these variables may have resulted in residual confounding, and future research should incorporate such psychosocial and clinical factors to provide a more comprehensive understanding of antenatal anxiety. In particular, the absence of objective sleep or actigraphy data limits our ability to explore mechanistic pathways linking nighttime smartphone use with anxiety through circadian disruption. Fifth, our analysis did not examine possible mediation or moderation effects—such as whether depressive symptoms mediate the relationship between smartphone overuse and anxiety, or whether the impact of smartphone use varies across gestational ages or education levels. This omission limits the ability to capture complex pathways underlying antenatal anxiety. Structural equation modeling or moderated mediation analysis may provide more nuanced insights in future studies. While our predictive model demonstrated good discrimination (AUC = 0.803) and calibration, its external validity remains untested. The model’s applicability to diverse populations (e.g., low-literacy or rural cohorts) and its performance in clinical settings outside of structured research environments require further evaluation. Moreover, smartphone use patterns are rapidly evolving with technology trends, and the behavioral variables used here may not remain stable over time. Finally, cultural specificity must be acknowledged: smartphone use patterns, social media engagement, and coping norms may vary substantially across sociocultural contexts. Therefore, our findings should be interpreted cautiously when extrapolated to Western or other cultural settings, where digital behaviors and anxiety correlates may differ. To illustrate the hypothesized mechanisms, we provide a conceptual model linking nighttime smartphone use, circadian and sleep disruption, and antenatal anxiety, with potential roles of depressive symptoms, education, social support, and hypertensive disorders of pregnancy (Supplementary Figure 1).

Clinically, our findings support the integration of brief smartphone use screening into routine antenatal care. For example, nurses may incorporate questions about daily screen time and nighttime usage into initial intake assessments, and women exceeding 4 h of daily use or using phones before bedtime could be flagged for mental health screening using GAD-7 or PHQ-9. The nomogram we developed offers a feasible tool for individualized anxiety risk prediction based on a combination of behavioral, psychological, and clinical data. Such approaches could be particularly valuable in resource-constrained settings where specialist mental health services are limited. In practical terms, clinicians could also provide psychoeducational counseling on healthy digital habits (e.g., limiting bedtime use, promoting “screen-free” routines), and digital tools such as app-based reminders or screen-time monitoring could be piloted as adjuncts. Importantly, these strategies should be adapted and validated in community and rural populations to ensure feasibility and cross-context applicability.

## Conclusion

Our findings indicate that smartphone overuse, nighttime use, social networking, depressive symptoms, hypertensive disorders of pregnancy, lower education level, and shorter gestational age are factors associated with antenatal anxiety. These associations highlight the potential value of integrating digital behavior monitoring and routine psychological screening into prenatal care, which may help identify women at elevated risk and inform targeted interventions to improve perinatal mental health outcomes.

## Data Availability

The raw data supporting the conclusions of this article will be made available by the authors, without undue reservation.

## Supplementary material

Supplementary Figure 1 Conceptual model linking nighttime smartphone use, sleep quality, and antenatal anxiety. This concept ual model illustrates the hypothesized pathways through which nighttime smartphone use may contribute to antenatal anxiety. Bedtime screen exposure is proposed to increase cognitive arousal and blue light–induced circadian disruption, leading to impaired sleep quality. These sleep disturbances, in turn, heighten vulnerability to anxiety. Depressive symptoms may act as a mediator, amplifying the impact of sleep disruption on anxiety. Sociodemographic and clinical factors such as education level, social support, and hypertensive disorders of pregnancy (HDP) may serve as moderators, influencing the strength of these associations. The model highlights potential mechanisms and contextual factors that should be examined in future longitudinal studies.
