# Supplementary figures and images for: Correlates of antenatal anxiety: smartphone use, depressive symptoms, and hypertensive disorders in a cross-sectional study in Southwest China
Source: Front Med (Lausanne). 2025 Oct 13;12:1682499. doi: 10.3389/fmed.2025.1682499 (PMC12554737; doi:10.3389/fmed.2025.1682499)

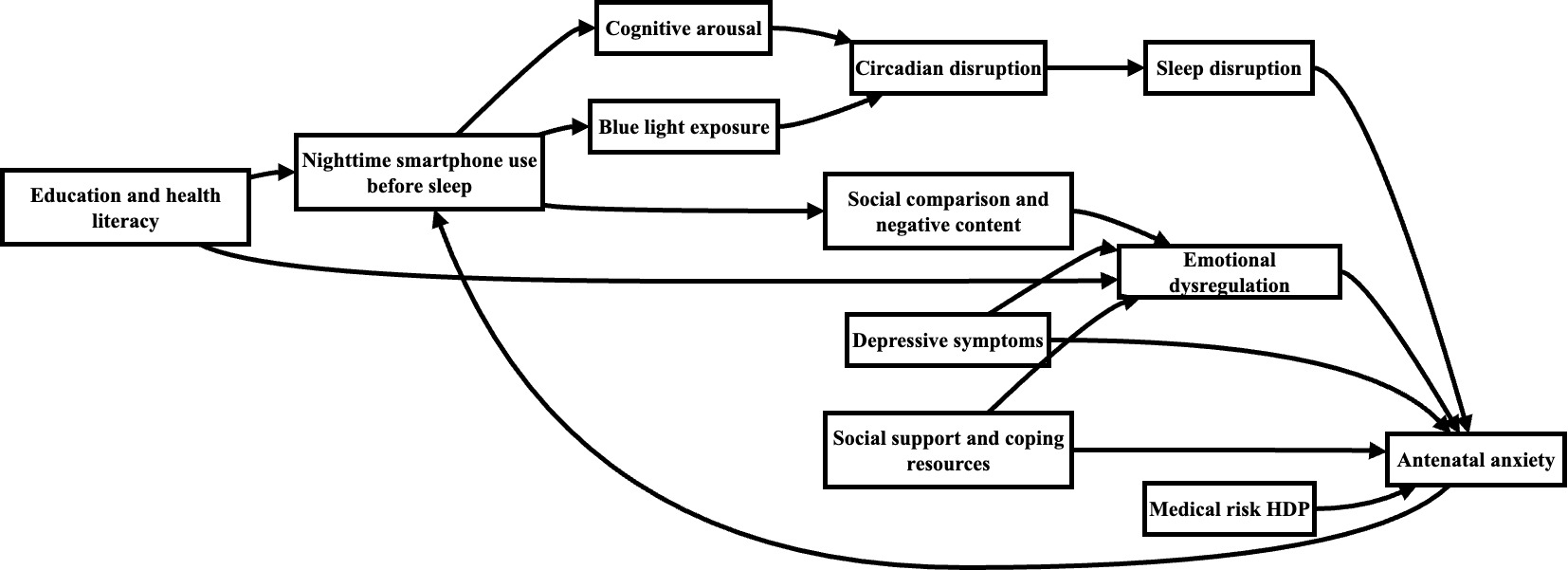

Supplement: Supplementary Figure 1 — Conceptual model linking nighttime smartphone use, sleep quality, and antenatal anxiety. This concept ual model illustrates the hypothesized pathways through which nighttime smartphone use may contribute to antenatal anxiety. Bedtime screen exposure is proposed to increase cognitive arousal and blue light–induced circadian disruption, leading to impaired sleep quality. These sleep disturbances, in turn, heighten vulnerability to anxiety. Depressive symptoms may act as a mediator, amplifying the impact of sleep disruption on anxiety. Sociodemographic and clinical factors such as education level, social support, and hypertensive disorders of pregnancy (HDP) may serve as moderators, influencing the strength of these associations. The model highlights potential mechanisms and contextual factors that should be examined in future longitudinal studies. [file Image_1.jpeg]
