# Supplementary material for: Correlates of antenatal anxiety: smartphone use, depressive symptoms, and hypertensive disorders in a cross-sectional study in Southwest China
Source: Front Med (Lausanne). 2025 Oct 13;12:1682499. doi: 10.3389/fmed.2025.1682499 (PMC12554737; doi:10.3389/fmed.2025.1682499)
Supplement: Supplementary file 2 [file Table_1.docx]

**Supplementary Table S1.** Sensitivity Analysis of Factors Associated with Antenatal Anxiety Using Alternative GAD-7 Cutoffs (≥8 vs ≥10)

| **Variable** | **Adjusted OR (95% CI), GAD-7 ≥10** | **P-value (≥10)** | **Adjusted OR (95% CI), GAD-7 ≥8** | **P-value (≥8)** |
| --- | --- | --- | --- | --- |
| Gestational age (per week increase) | 0.94 (0.91–0.98) | 0.002 | 0.95 (0.92–0.98) | 0.004 |
| Education: Junior high or below (vs college+) | 1.81 (1.12–2.91) | 0.015 | 1.75 (1.14–2.68) | 0.012 |
| Education: High school/technical (vs college+) | 1.24 (0.87–1.75) | 0.230 | 1.20 (0.86–1.66) | 0.265 |
| Hypertensive disorders of pregnancy (HDP) (vs none) | 2.06 (1.19–3.58) | 0.010 | 2.01 (1.21–3.35) | 0.008 |
| Daily smartphone use: 2–4 h (vs <2 h) | 1.49 (1.00–2.25) | 0.049 | 1.44 (0.99–2.10) | 0.056 |
| Daily smartphone use: >4 h (vs <2 h) | 3.01 (1.95–4.66) | <0.001 | 2.92 (1.89–4.50) | <0.001 |
| Nighttime smartphone use (yes vs no) | 2.26 (1.58–3.23) | <0.001 | 2.19 (1.54–3.12) | <0.001 |
| Main use: social networking (vs information seeking) | 1.57 (1.10–2.23) | 0.013 | 1.53 (1.08–2.19) | 0.015 |
| PHQ-9 score (per 1-point increase) | 1.31 (1.23–1.39) | <0.001 | 1.29 (1.22–1.37) | <0.001 |

**Notes:** Results are from multivariate logistic regression models adjusting for all listed covariates. Anxiety was defined as GAD-7 ≥10 (main analysis, Table 3) and GAD-7 ≥8 (sensitivity analysis). OR = odds ratio; CI = confidence interval; HDP = hypertensive disorders of pregnancy.
